# Predicting Observable Quantities of Self-Assembled Metamaterials from the T-Matrix of Its Constituting Meta-Atom

**DOI:** 10.3390/ma11020213

**Published:** 2018-01-30

**Authors:** Radius N. S. Suryadharma, Carsten Rockstuhl

**Affiliations:** 1 Institute of Theoretical Solid State Physics, Karlsruhe Institute of Technology, Wolfgang Gaede Str. 1, 76131 Karlsruhe, Germany; carsten.rockstuhl@kit.edu; 2 Institute of Nanotechnology, Karlsruhe Institute of Technology, P.O. Box 3640, 76021 Karlsruhe, Germany

**Keywords:** metamaterials, self-assembled, photonics, scattering, chirality, plasmonics

## Abstract

Self-assembled metamaterials attract considerable interest as they promise to make isotropic bulk metamaterials available at low costs. The optical response of self-assembled metamaterials is derived predominantly from the response of its individual constituents, i.e., the meta-atoms. Beyond effective properties, primary experimentally observable quantities, such as specific cross-sections, are at the focus of interest as they are frequently considered when exploiting metamaterials in specific applications. This posses the challenge of predicting these observable quantities for a diluted ensemble of randomly oriented meta-atoms. Thus far, this has been achieved by either averaging the optical response of the meta-atom across all possible incident fields or by restricting the consideration to only an electric and magnetic dipolar response. This, however, is either time-consuming or imposes an unnecessary limitation. Here, we solve this problem by deriving and presenting explicit expressions for experimentally observable quantities of metamaterials made from randomly arranged and oriented meta-atoms characterized by their T-matrix.

## 1. Introduction

Metamaterials are artificial materials that exhibit unique properties not encountered in nature. The properties of metamaterials are largely derived from the scattering properties of its constituents, the so called meta-atoms. The recent advances in the field of metamaterials has opened up many unprecedented means of light manipulation [1,2,3,4,5,6]. These advances were only possible thanks to meta-atoms that do not just show an electric but also a magnetic response [7,8,9]. Moreover, the electro-magnetic response can be cross-coupled [10,11], giving rise to a plethora of phenomena for circular polarization states of light [12,13,14,15].

In the initial stage of metamaterials development, especially in the optical regime, the majority of experimental realizations were made with top-down nanofabrication techniques such as lithography techniques or electron beam writing [16,17,18,19]. These methods are reliable and offer a high degree of spatial control in two dimensions. However, they hardly enable truly three-dimensional materials. To mitigate this limitation, bottom-up approaches that combine nanochemistry and colloidal physics were proposed [20,21]. They allow to easily produce 3D metamaterials with isotropic properties at low costs [22,23,24,25,26].

With the improved understanding of meta-atoms, it became soon appreciated that theoretically considering metamaterials only in terms of effective properties is a somewhat limited point-of-view [27,28]. Instead, the consideration of metamaterials as an ensemble of complicated scatterers with a response that can be tailored (largely) will unlock quite a lot of exciting applications [29]. It requires to consider the response not just in terms of an induced electric and magnetic dipole moment, but also quadrupolar or octopolar multipole moments are taken into account. In general, any arbitrary higher order multipole moment can be studied [30]. These induced moments are linked to the expansion coefficients of the incident field by the T-matrix [31]. With that, the T-matrix constitutes in essence the aggregated information that expresses how an object interacts with any possible illumination. Knowing the scattering object means knowing the T-matrix [32].

Exploiting the coherent interference among different multipolar orders in their contribution to observable properties is at the heart of many envisioned applications. Examples are the spectrally broadband suppression of backscattering of pretty large low-index spheres [33] or helicity filtering glasses made from maximal electromagnetic chiral scatterers [34]. The analysis of all such applications requires the ability to predict experimentally observable quantities for an ensemble of randomly oriented and randomly arranged meta-atoms. Observable quantities of interest are, e.g., the absorption cross-section.

While considering a sufficiently diluted ensemble of meta-atoms where nearest neighbour interaction is negligibly small, only for the special case of isotropic meta-atoms the response of the ensemble can be directly deduced from the response of the constituent [35,36]. For a sufficiently small particle that justifies a consideration in the dipolar regime, observable quantities have been expressed in term of respective polarizabilities [37]. For an arbitrarily shaped meta-atom, the response has been deduced thus far by averaging the response of the individual meta-atom across all possible orientations of the meta-atom relative to the illumination [38,39]. This can be an extremely tedious task, as quite some quantities of interest show a very poor convergence with respect to the considered number of illuminations, as we will show later in the article.

To solve this problem, we develop here a methodological framework that allows to predict experimentally observable quantities of sufficiently diluted self-assembled metamaterials directly from the T-matrix of their constituent. Comparison to predictions of the same quantities obtained from an averaging of the response to randomly chosen incident fields demonstrate the liability of our expressions. We emphasize that some observable quantities show a poor convergence for the latter approach and require hence a large number of illumination scenarios to be considered in the averaging. Here, our approach clearly improves the situation. Exemplarily calculations to demonstrate the strength of our methodology are equally documented in this contribution. We concentrate on the example of a helical arrangement of metallic nanoparticles; being an ensemble that unifies the beauty of possessing a dispersive response in all the quantities we are interested in and being of practical relevance.

## 2. T-Matrix Formalism

Solving the electromagnetic scattering problem requires to link a specific incident field to the scattered field. The details of this process are governed by the shape and the material from which the scatterer is made. Here, we only require the scatterer to be localized in space. In general, the incident and scattered fields of any given object can be decomposed into an orthogonal set of basis vectors. For our purpose, it is most convenient to use vector spherical wave functions in parity [$M_{\mathrm{lmn}}\left(r,\theta ,\phi ,\omega \right)$] or helicity [$M_{\mathrm{hmn}}\left(r,\theta ,\phi ,\omega \right)$] bases. Throughout the paper, we will use subscript l to denote parity basis and h for the helicity basis. By employing vector spherical wave functions as the basis set, the incident time harmonic electric field oscillating at a fixed frequency ω can be expanded as [40]

(1) $$E_{\mathrm{inc}}\left(r,\theta ,\phi ,\omega \right)=\sum_{j=1}^{2}\sum_{n=1}^{N}\sum_{m=-n}^{n}p_{\mathrm{jnm}}\left(\omega \right)M_{\mathrm{jnm}}^{\left(1\right)}\left(r,\theta ,\phi ,\omega \right),$$

While the electric field scattered by any given object reads as:

(2) $$E_{\mathrm{sca}}\left(r,\theta ,\phi ,\omega \right)=\sum_{j=1}^{2}\sum_{n=1}^{N}\sum_{m=-n}^{n}a_{\mathrm{jnm}}\left(\omega \right)M_{\mathrm{jnm}}^{\left(3\right)}\left(r,\theta ,\phi ,\omega \right).$$

Here, $M_{\mathrm{jnm}}^{\left(i\right)}\left(r,\theta ,\phi ,\omega \right)$ refers to the vector spherical wave functions, $p_{\mathrm{jnm}}$ and $a_{\mathrm{jnm}}$ are the expansion coefficients, indices n and m refer to quantum numbers, and index j refers to the parity (j=l) or the helicity (j=h) of the vector spherical harmonics [34]. The superscript in the vector spherical harmonics denotes the choice of either the spherical Bessel function (1) or the outgoing spherical Hankel function (3) as the argument of the function. To simplify our notation, we drop from now on the frequency dependency. However, it is implicitly assumed throughout the paper.

The T-matrix $\bar{\bar{T}}$ links the incident and scattered field coefficients of any given object. The T-matrix is an inherent property of the object and does not depend on the illumination. In the T-matrix formulation, the scattered and incident fields coefficients can be linked as [41]:

(3) $$\bar{\bar{T}}p=a.$$

Here, p and a are vectors that are composed of the respective linear coefficients $p_{\mathrm{jnm}}$ and $a_{\mathrm{jnm}}$, respectively. Please note, the T-matrix for an arbitrary object can be obtained from numerically computing the response of the scatterer to a multipolar illumination combined with the proper calculation of the induced multipole moments [32]. Also, particularly for self-assembled metamaterials made from an ensemble of spherical objects, effective routines exists to construct the T-matrix of the object from the T-matrices of the individual constituents [42]. In the following, we will assume that the T-matrix is known for a given scatterer. Sometimes, it is more convenient to spell out the parity or helicity of the incident and scattered fields in the T-matrix formulation. In the parity basis it reads as

(4) $$\left(\begin{array}{cc} {\bar{\bar{T}}}_{\mathrm{ee}} & {\bar{\bar{T}}}_{\mathrm{em}}\\ {\bar{\bar{T}}}_{\mathrm{me}} & {\bar{\bar{T}}}_{\mathrm{mm}} \end{array}\right)\left(\begin{array}{c} p_{l=1}\\ p_{l=2} \end{array}\right)=\left(\begin{array}{c} a_{l=1}\\ a_{l=2} \end{array}\right).$$

Here, the parity index refers to electric (l=1) or magnetic (l=2) multipolar contributions, respectively. The sub-matrices ${\bar{\bar{T}}}_{\mathrm{em}}$ and ${\bar{\bar{T}}}_{\mathrm{me}}$ describe the coupling of electric and magnetic multipolar moments of the object, while the sub-matrices ${\bar{\bar{T}}}_{\mathrm{ee}}$ and ${\bar{\bar{T}}}_{\mathrm{mm}}$ describe the coupling of it’s electric-electric or magnetic-magnetic multipolar components. On the other hand, by using the helicity basis, the T-matrix reads as

(5) $$\left(\begin{array}{cc} {\bar{\bar{T}}}_{\mathrm{LL}} & {\bar{\bar{T}}}_{\mathrm{LR}}\\ {\bar{\bar{T}}}_{\mathrm{RL}} & {\bar{\bar{T}}}_{\mathrm{RR}} \end{array}\right)\left(\begin{array}{c} p_{h=1}\\ p_{h=2} \end{array}\right)=\left(\begin{array}{c} a_{h=1}\\ a_{h=2} \end{array}\right),$$

where the helicity index refers to left (h=1) or right (h=2) handed circularly polarized (RCP and LCP) fields, respectively. Both T-matrix formulation can be linked by:

(6) $$\left(\begin{array}{cc} {\bar{\bar{T}}}_{\mathrm{LL}} & {\bar{\bar{T}}}_{\mathrm{LR}}\\ {\bar{\bar{T}}}_{\mathrm{RL}} & {\bar{\bar{T}}}_{\mathrm{RR}} \end{array}\right)=\frac{1}{2}\left(\begin{array}{cc} \bar{\bar{I}} & \bar{\bar{I}}\\ \bar{\bar{I}} & -\bar{\bar{I}} \end{array}\right)\left(\begin{array}{cc} {\bar{\bar{T}}}_{\mathrm{ee}} & {\bar{\bar{T}}}_{\mathrm{em}}\\ {\bar{\bar{T}}}_{\mathrm{me}} & {\bar{\bar{T}}}_{\mathrm{mm}} \end{array}\right)\left(\begin{array}{cc} \bar{\bar{I}} & \bar{\bar{I}}\\ \bar{\bar{I}} & -\bar{\bar{I}} \end{array}\right),$$

where $\bar{\bar{I}}$ is the identity matrix with the same size as the sub-matrix ${\bar{\bar{T}}}_{\mathrm{LL}}$.

## 3. Predicting Observable Quantities from the T-Matrix of Individual Meta-Atoms

In this section, we will show how experimentally observable quantities of a bulk self-assembled metamaterial can be deduced from its individual constituents. For this purpose, we assume that there is no interaction between individual meta-atom that would cause a renormalization of the respective T-matrix. It requires the distances between the meta-atoms to be sufficiently large. All meta-atoms are randomly oriented inside a solution and the number of particles is very large. Based on these assumptions, an ensemble averaging will be employed and experimentally observable quantities can be deduced from the T-matrix of the individual meta-atom. Since the total response of bulk self-assembled metamaterials is equivalent to the averaged response from its individual meta-atom, the properties of bulk metamaterials can be inferred from the average response of an individual meta-atom. This will be the starting point of our analysis. For the sake of brevity, we will only present the derivation of the average scattering cross section of such ensemble of meta-atoms in detail. The average of other parameters will only be listed in Table 1 but can be derived analogously.

For a particular illumination k, which we think of as a plane wave propagating in a random direction and having a random polarization, the scattering cross section ($\sigma_{\mathrm{sca}}^{k}$) can be written as:

(7) $$\sigma_{\mathrm{sca}}^{k}=\frac{4\pi }{k_{b}^{2}}\sum_{l=1}^{2}\sum_{n=1}^{N}\sum_{m=-n}^{n}\left|a_{\mathrm{lnm}}^{k}\right|2,$$

where index k denotes the indices for a particular illumination k and $k_{b}$ denotes wave number inside the background medium, which is defined as $k_{b}=\frac{\omega }{c}$, with *c* being the speed of light in the background medium. Writing it in Dirac bra-ket notation it reads as

(8) $$\sigma_{\mathrm{sca}}^{k}=\frac{4\pi }{k_{b}^{2}}\langle a^{k}|a^{k}\rangle ,$$

where $a^{k}$ is a column vector which contains the parameter $a_{\mathrm{lnm}}^{k}$. By employing the T-matrix definition as written in Equation (3), we arrive at:

(9) $$\sigma_{\mathrm{sca}}^{k}=\frac{4\pi }{k_{b}^{2}}\langle p^{k}\left|{\bar{\bar{T}}}^{†}\bar{\bar{T}}\right|p^{k}\rangle .$$

In the next step we reformulate the inner product as described in Equation (9) as the trace of an outer product of a matrix. With that we can write $\sigma_{\mathrm{sca}}^{k}$ as:

(10) $$\sigma_{\mathrm{sca}}^{k}=\frac{4\pi }{k_{b}^{2}}\mathrm{Tr}\left[{\bar{\bar{T}}}^{†}\bar{\bar{T}}|p^{k}\rangle \langle p^{k}|\right],$$

where Tr[] denotes the trace of a matrix. Rewriting the above equation in compact form we arrive at

(11) $$\sigma_{\mathrm{sca}}^{k}=\frac{4\pi }{k_{b}^{2}}\mathrm{Tr}\left[{\bar{\bar{T}}}^{†}{\bar{\bar{T}}}^{}{\bar{\bar{X}}}^{k}\right],$$

where ${\bar{\bar{X}}}^{k}=|p^{k}\rangle \langle p^{k}|$. The above equation expresses the scattering cross-section for a specific illumination in matrix form. By taking the average across multiple illumination scenarios, the following equation will hold:

(12) $$\sigma_{\mathrm{sca}}^{\mathrm{aver}}=\frac{4\pi }{k_{b}^{2}}\frac{1}{K}\sum_{k=1}^{K}\mathrm{Tr}\left[{\bar{\bar{T}}}^{†}{\bar{\bar{T}}}^{}{\bar{\bar{X}}}^{k}\right]=\frac{4\pi }{k_{b}^{2}}\mathrm{Tr}\left[{\bar{\bar{T}}}^{†}\bar{\bar{T}}\frac{1}{K}\sum_{k=1}^{K}{\bar{\bar{X}}}^{k}\right].$$

Since the vector matrix $|p^{k}\rangle$ is a normalized vector composed of the expansion coefficients of the incident field, for a random polarization scenario, this vector will be a random, normalized vector. Using this fact and assuming that K is a very large number, the following relation will hold

(13) $$\frac{1}{K}\sum_{k=1}^{K}{\bar{\bar{X}}}^{k}=\frac{1}{S}\bar{\bar{I}},$$

where $\bar{\bar{I}}$ is the identity matrix and *S* is the dimension of the vector $|p^{k}\rangle$. Using this fact, the average scattering cross section can be written as:

(14) $$\sigma_{\mathrm{sca}}^{\mathrm{aver}}=\frac{4\pi }{Sk_{b}^{2}}\mathrm{Tr}\left[{\bar{\bar{T}}}^{†}\bar{\bar{T}}\right].$$

Following the same derivation, explicit expressions for other averaged observable properties can be derived. Table 1 summarizes relevant averaged quantities that can be extracted from the T-matrix of the meta-atom directly.

So far, the average parameters are defined as an average across random illumination directions and polarizations. This approach, while quite useful for many cases, is however unable to predict quantities that arise from an illumination of the metamaterial with light of a specific polarization. For example, measuring the chiral properties of self-assembled metamaterials requires a distinction of the response to either left or right handed circularly polarized light. To extend the approach described above to accommodate this requirement, it is therefore necessary to use the helicity based T-matrix. Then, the expansion coefficient of the incident field in Equation (5) can be written as

(15) $$p^{L}=\left(\begin{array}{c} p_{h=1}\\ 0 \end{array}\right),$$

or

(16) $$p^{R}=\left(\begin{array}{c} 0\\ p_{h=2} \end{array}\right).$$

The superscripts R or L denote right or left handed circularly polarized incident field, respectively. Since the incident field is always written as a normalized vector, it implies that either $p_{1}$ or $p_{2}$ is a normalized vector. By inserting the above equations into Equation (5), the average values of several parameters for a particular polarization can be derived, as summarized in Table 2. Note that the circular dichroism is defined as the difference of the attenuation coefficients between RCP and LCP. In this context, the relation between attenuation coefficient and absorption cross section needs to be applied. This relation is:

(17) $$\alpha^{\mathrm{pol}}=M\sigma_{\mathrm{abs}}^{\mathrm{pol}}$$

where α is the attenuation coefficient, *M* is the particle density per unit volume and $\sigma_{\mathrm{abs}}$ denotes the absorption cross section. The superscript pol denotes the polarization of the incident field. Note that, the absorption cross section for different polarization scenarios was already previously derived albeit in a different way [34].

## 4. Results

In this section, we will discuss the implementation of the formulas listed in Table 1 and Table 2 and compare it with the averaging method. To demonstrate the applicability, we consider a meta-atom consisting of ten gold nanoparticles with 80 nm radius. The gold permittivity is taken from literature [43] and these nanoparticles are arranged in a helical structure with a total height of 1200 nm and 200 nm radius. The helix consists of two pitches and the distance among neighbouring gold nanoparticles along the helix is identical. We assume that the meta-atom is immersed in water (with background refractive index, $n_{b}=1.33$). To achieve a good convergence, the expansion order for the T-matrix is taken as N=4. The T-matrix was calculated using the algorithm described in literature [42].

**Figure 1:**
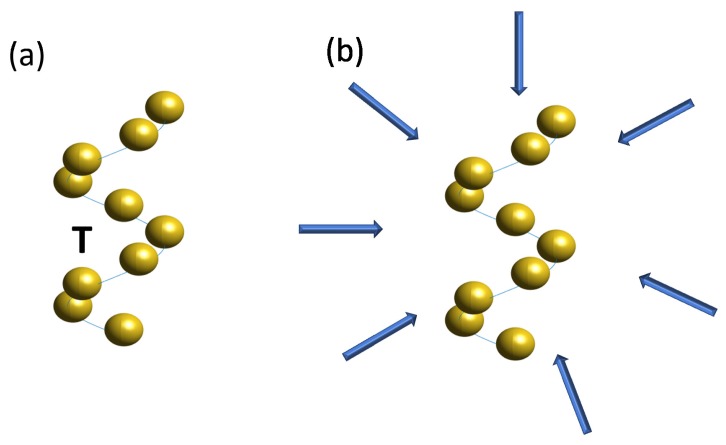
Illustration of the difference between (**a**) T-matrix extraction procedure and (**b**) averaging procedure. Here, the averaging procedure is done using several plane waves with different direction of propagation. The considered meta-atom consists of a helical arrangement of gold nanoparticles with a radius of 80 nm arranged in a helix. The helix has a total height 1200 nm and a radius 200 nm. The helix consist of 2 pitches.

**Figure 2:**
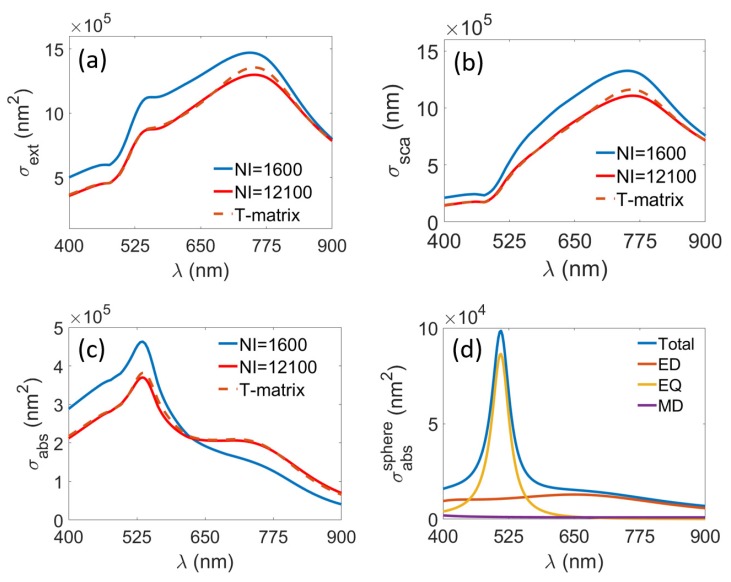
Average (**a**) extinction; (**b**) scattering; and (**c**) absorption cross section of gold nanospheres arranged in a helix. The blue straight lines denote the value extracted from manual averaging method with 1600 number of illumination (NI), the red straight lines using 12,100 NI, while the brown dashed lines are extracted directly from the T-matrix, as described in Table 1; and (**d**) absorption cross section of single gold sphere with radius 80 nm and it’s multipolar decomposition. ED, EQ and MD denote electric dipole, electric quadrupole and magnetic dipole, respectively.

The averaging method will be done using two different number of illuminations (NI), 1600 and 12,100. The incident fields are always plane waves with random direction of propagation, as shown in Figure 1. Here, we use a random number to generate two parameters, the polar (θ) and the azimuthal (ϕ) angles. The polar angle can be any real number between $-\frac{\pi }{2}$ and $\frac{\pi }{2}$, while the azimuthal angle ϕ can be any real number between 0 and 2π. Next, we define several set of pairs comprising a specific θ and a specific ϕ. The wave vector of the incident field k then can be defined from these two parameters as $k=\left[k_{x}\;k_{y}\;k_{z}\right]=k_{b}\left[\sin\theta \cos\phi \;\sin\theta \sin\phi \;\cos\theta \right]$. The contribution from each plane wave is then weighted with the factor of cosθ. To validate our approach, we compare the extinction, scattering, and absorption cross sections averaged across the random illuminations (as discussed in Table 1) and compare it with predictions that rely explicitly only on the T-matrix. Comparative results are shown in Figure 2.

Figure 2a shows the average extinction cross section of the meta-atom described in Figure 1. From both methods (manual averaging and parameters extraction from the T-matrix), two distinct peaks can be observed, one around 548 nm and the other one at 743 nm. These two peaks correspond roughly to the resonance positions of individual gold nanoparticle, which resonates at 542 nm (electric quadrupole mode) and 673 nm(electric dipole mode). The resonance shift from these values can be attributed to the coupling between the nanoparticle and it’s adjacent neighbours in the helical structure. The same agreement between both methods is also observed in Figure 2b,c, where the scattering and absorption cross sections are shown. Here, the resonance peaks also match perfectly. It can also be seen directly that only for the larger number of plane waves (NI) considered in the averaging procedure, the predicted values almost agree with those obtained from the parameter extracted directly from the T-matrix, suggesting that for a very large number of plane waves, both methods will arrive at the same value. This suggests that the parameter extraction from the T-matrix offers a powerful and fast way to calculate the responses of the ensemble of meta-atoms [32]. On the other hand, Figure 2d shows the absorption cross section of single gold nanosphere and its decomposition into its multipolar component, where two distinct peaks around 511 nm and 648 nm can be attributed to electric quadrupole and electric dipole moments of the sphere, respectively. It can be observed that the resonance peaks of helical structure become broader compared to the resonance positions of individual gold nanosphere due to the coupling between gold nanoparticles.

**Figure 3:**
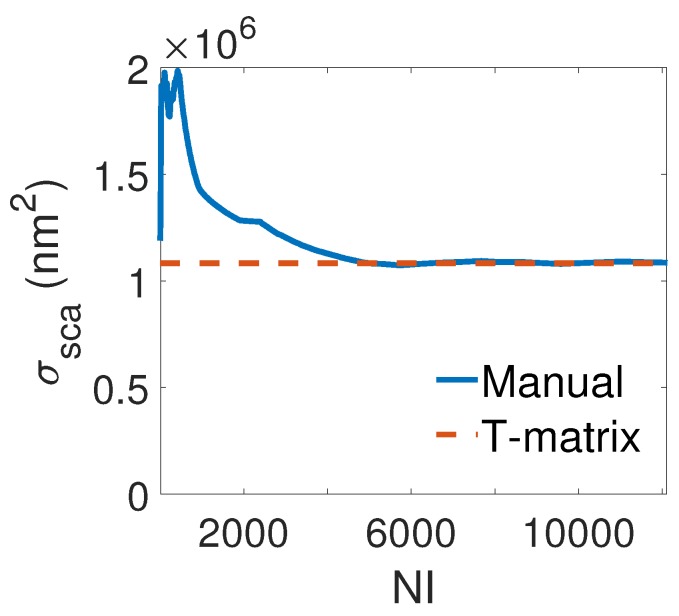
Convergence of scattering cross section with respect to the number of illumination of the gold nanospheres arranged in a helix for the wavelength of 730 nm. The brown dashed lines denote the value extracted directly from the T-matrix while the blue straight lines denote the value obtained from averaging method for different number of illumination directions.

To validate the convergence of manual averaging method, we compare the value for the average scattering cross section obtained once directly from the T-matrix and once by averaging the response across an increasingly larger number of randomly chosen illumination directions. Results of these quantities as a function of the number of considered illumination scenarios is shown in Figure 3. As it can be clearly seen, the value obtained from both methods reach the same value for NI > 5000.

**Figure 4:**
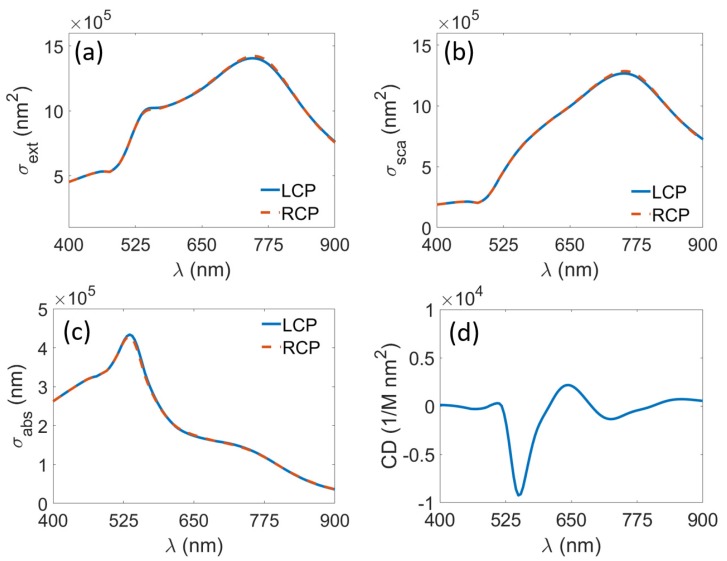
Average (**a**) extinction; (**b**) scattering and (**c**) absorption cross section of gold nanospheres arranged in a helix for different polarization of incident field. The brown dashed lines denote right handed polarization, while the blue straight lines denote the left handed polarization and (**d**) circular dichroism.

So far, we only discussed the results for a randomly polarized light. This approach, however, wil not be able to extract polarization dependent effects from our self-assembled metamaterials, such as circular dichroism. For this purpose, we will employ the equations shown in Table 2, as depicted in Figure 4. Here, both polarizations have almost the same response, due to the fact that the extinction, scattering, and absorption are measure of power, which does not strongly depend on the polarization of the incident field. However, from the tiny differences another important parameter for circularly polarized light can be extracted, that is, the circular dichroism (CD). Using the parameter extraction from the T-matrix enables us to calculate this parameter exactly and beyond the dipole approximation. Figure 4d shows the CD obtained using N=4. The strongest CD signal can be observed around 538 nm and several smaller peaks or dips around 645 nm, 510 nm, and 716 nm. The CD around 538 nm has the strongest signal due to the fact that the strongest absorption of the individual gold nanoparticle happens to be around 511 nm, as observed in Figure 2d. Due to this fact, the coupling between gold nanoparticles will be strongest around this wavelength, and by extension, the general response (CD in this case). Here, it can be inferred that the CD peaks at 538 nm and 645 nm related to electric quadrupole and electric dipole of individual gold nanoparticle, respectively. Depending on the chosen material and the size of the particle, these wavelengths can be tuned. Here, we show that the parameters extraction from the T-matrix of meta-atom offers a reliable and convenient way to analyze the response of the bulk ensemble of diluted self-assembled metamaterials without the need to do manual averaging procedure.

## 5. Conclusions

In conclusion, we have shown that, for a sufficiently diluted self-assembled metamaterial, experimentally observable properties of the bulk material can be directly calculated once the T-matrix of the individual meta-atom is known. With that, our approach circumvent the necessity to manually average the response across a larger number of different illumination scenarios. Finally, the extraction of polarization dependent parameters was also presented, where the CD response can be calculated efficiently and well beyond the dipole approximation. Here, we provide a comfortable and direct way to calculate the responses of diluted self-assembled metamaterials without the need to do the averaging procedure. This work may provide impetus of designing the responses of self assembled metamaterials based on their meta-atom in a fast and reliable way. Also, the analysis of more general disordered amorphous photonic structures will benefit from our contribution.

## Figures and Tables

**Table 1:** Mathematical expression for a particular illumination as well as the average value extracted from the parity based T-matrix of the meta-atom for various parameters.

No.	Name of Parameter	Expression for One Particular Illumination	Average Value
1	Scattering cross section	$\sigma_{\mathrm{sca}}=\frac{4\pi }{k_{b}^{2}}\sum_{l=1}^{2}\sum_{n=1}^{N}\sum_{m=-n}^{n}{\left|a_{\mathrm{lnm}}\right|}^{2}$	$\sigma_{\mathrm{sca}}^{\mathrm{aver}}=\frac{4\pi }{Sk_{b}^{2}}\mathrm{Tr}\left[{\bar{\bar{T}}}^{†}\bar{\bar{T}}\right]$
2	Extinction cross section	$\sigma_{\mathrm{ext}}=\frac{4\pi }{k_{b}^{2}}ℜ\sum_{l=1}^{2}\sum_{n=1}^{N}\sum_{m=-n}^{n}p_{\mathrm{lnm}}^{*}a_{\mathrm{lnm}}$	$\sigma_{\mathrm{ext}}^{\mathrm{aver}}=\frac{4\pi }{Sk_{b}^{2}}ℜ\mathrm{Tr}\left[\bar{\bar{T}}\right]$
3	Absorption cross section	$\sigma_{\mathrm{abs}}=\sigma_{\mathrm{ext}}-\sigma_{\mathrm{sca}}$	$\sigma_{\mathrm{abs}}^{\mathrm{aver}}=\frac{4\pi }{Sk_{b}^{2}}ℜ\mathrm{Tr}\left[\bar{\bar{T}}\left(\bar{\bar{I}}-{\bar{\bar{T}}}^{†}\right)\right]$

**Table 2:** Average value of several responses for Left Circularly Polarized (LCP) and Right Circularly Polarized (RCP) light in terms of the components of the helicity based T-matrix of the individual meta-atom.

No	Parameter	Average Value
1	Scattering cross section (LCP)	$\frac{4\pi }{Sk_{b}^{2}}\mathrm{Tr}\left[{\bar{\bar{T}}}_{\mathrm{LL}}^{†}{\bar{\bar{T}}}_{\mathrm{LL}}+{\bar{\bar{T}}}_{\mathrm{LR}}^{†}{\bar{\bar{T}}}_{\mathrm{LR}}\right]$
2	Extinction cross section (LCP)	$\frac{4\pi }{Sk_{b}^{2}}ℜ\mathrm{Tr}\left[{\bar{\bar{T}}}_{\mathrm{LL}}\right]$
3	Absorption cross section (LCP)	$\frac{4\pi }{Sk_{b}^{2}}ℜ\mathrm{Tr}\left[{\bar{\bar{T}}}_{\mathrm{LL}}\left(\bar{\bar{I}}-{\bar{\bar{T}}}_{\mathrm{LL}}^{†}\right)-{\bar{\bar{T}}}_{\mathrm{LR}}^{†}{\bar{\bar{T}}}_{\mathrm{LR}}\right]$
4	Scattering cross section (RCP)	$\frac{4\pi }{Sk_{b}^{2}}\mathrm{Tr}\left[{\bar{\bar{T}}}_{\mathrm{RR}}^{†}{\bar{\bar{T}}}_{\mathrm{RR}}+{\bar{\bar{T}}}_{\mathrm{RL}}^{†}{\bar{\bar{T}}}_{\mathrm{RL}}\right]$
5	Extinction cross section (RCP)	$\frac{8\pi }{Sk_{b}^{2}}ℜ\mathrm{Tr}\left[{\bar{\bar{T}}}_{\mathrm{RR}}\right]$
6	Absorption cross section (RCP)	$\frac{4\pi }{Sk_{b}^{2}}ℜ\mathrm{Tr}\left[{\bar{\bar{T}}}_{\mathrm{RR}}\left(\bar{\bar{I}}-{\bar{\bar{T}}}_{\mathrm{RR}}^{†}\right)-{\bar{\bar{T}}}_{\mathrm{RL}}^{†}{\bar{\bar{T}}}_{\mathrm{RL}}\right]$
7	Circular Dichroism	$\frac{4M\pi }{Sk_{b}^{2}}ℜ\mathrm{Tr}[{\bar{\bar{T}}}_{\mathrm{LL}}\left(\bar{\bar{I}}-{\bar{\bar{T}}}_{\mathrm{LL}}^{†}\right)-{\bar{\bar{T}}}_{\mathrm{RR}}\left(\bar{\bar{I}}-{\bar{\bar{T}}}_{\mathrm{RR}}^{†}\right)]$
